# Supplementary material for: Molecular Characterization and Heterologous Production of the Bacteriocin Peocin, a DNA Starvation/Stationary Phase Protection Protein, from Paenibacillus ehimensis NPUST1
Source: Molecules. 2019 Jul 9;24(13):2516. doi: 10.3390/molecules24132516 (PMC6650805; doi:10.3390/molecules24132516)

F1 Sequence: EAEGEESAER, Charge: +2, Monoisotopic m/z: 553.73364 Da (+0.15 mmu/+0.28 ppm), MH+: 1106.46001 Da, RT: 17.18 min,
Identified with: Mascot (v1.30); IonScore:77, Exp Value:1.1E-008, Ions matched by search engine: 9/88

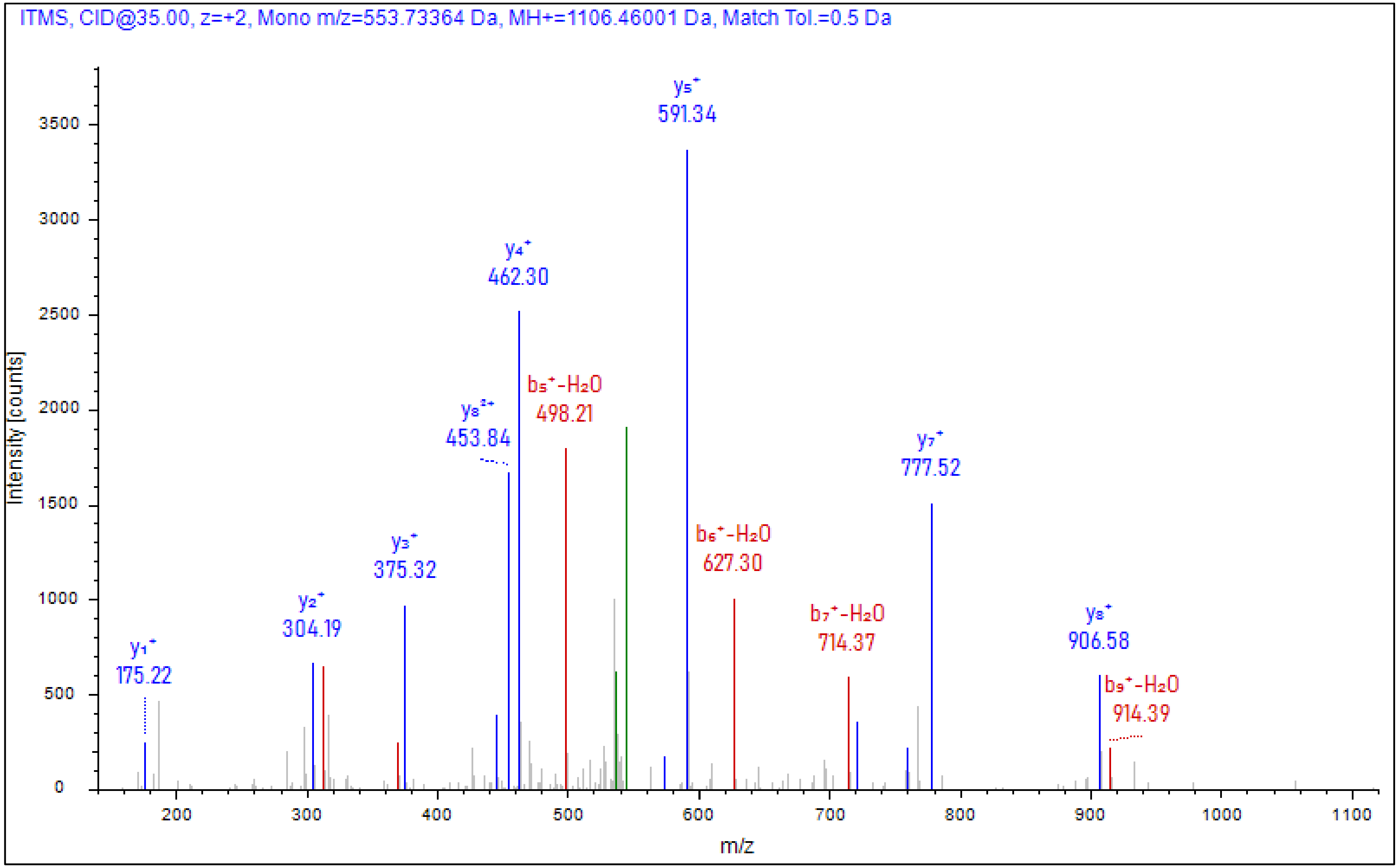

F2 Sequence: GPQFFTLHTK, Charge: +2, Monoisotopic m/z: 588.31396 Da (-0.08 mmu/-0.13 ppm), MH+: 1175.62065 Da, RT: 38.20 min, Identified with: Mascot (v1.30); IonScore:62, Exp Value:1.5E-006, Ions matched by search engine: 14/92

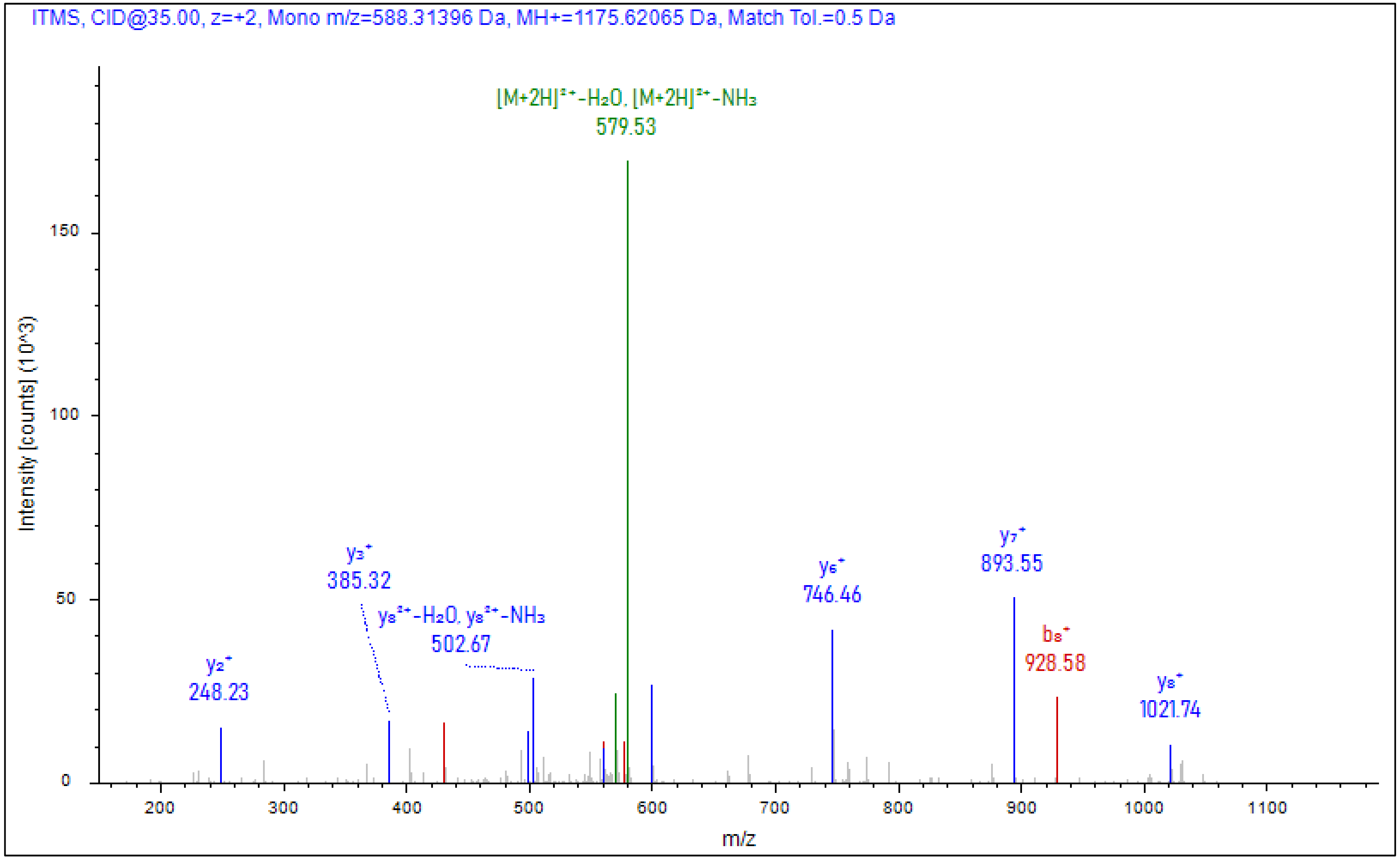

F3 Sequence: LHNYHWYVK, Charge: +2, Monoisotopic m/z: 630.31903 Da (-0.61 mmu/-0.97 ppm), MH+: 1259.63078 Da, RT: 66.67 min, Identified with: Mascot (v1.30); IonScore:45, Exp Value:6.5E-005, Ions matched by search engine: 8/78

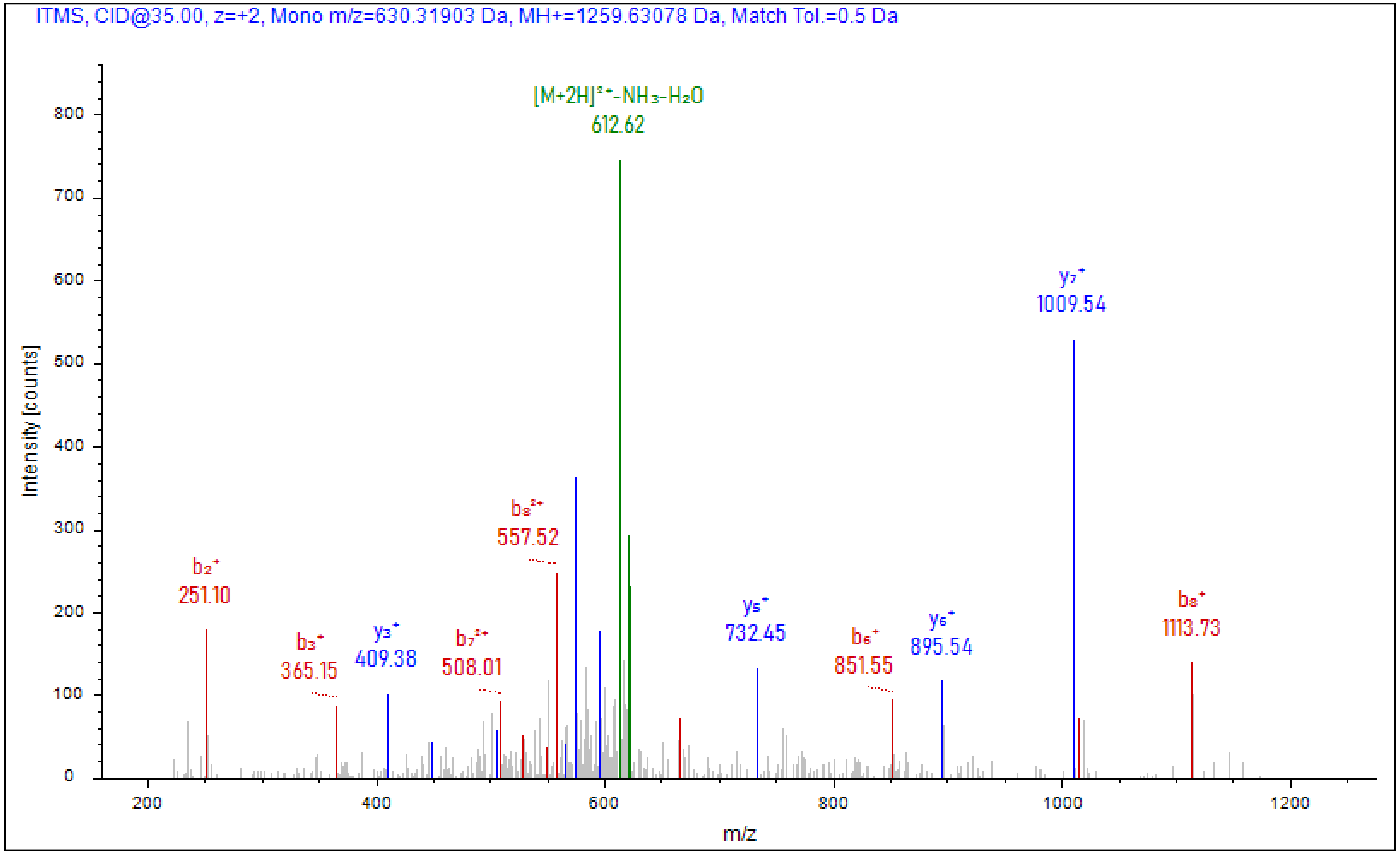

F4 Sequence: HVWMLNAFLGN, Charge: +2, Monoisotopic m/z: 651.32672 Da (+0.08 mmu/+0.13 ppm), MH+: 1301.64617 Da, RT: 59.59 min, Identified with: Mascot (v1.30); IonScore:60, Exp Value:2.1E-006, Ions matched by search engine: 9/92

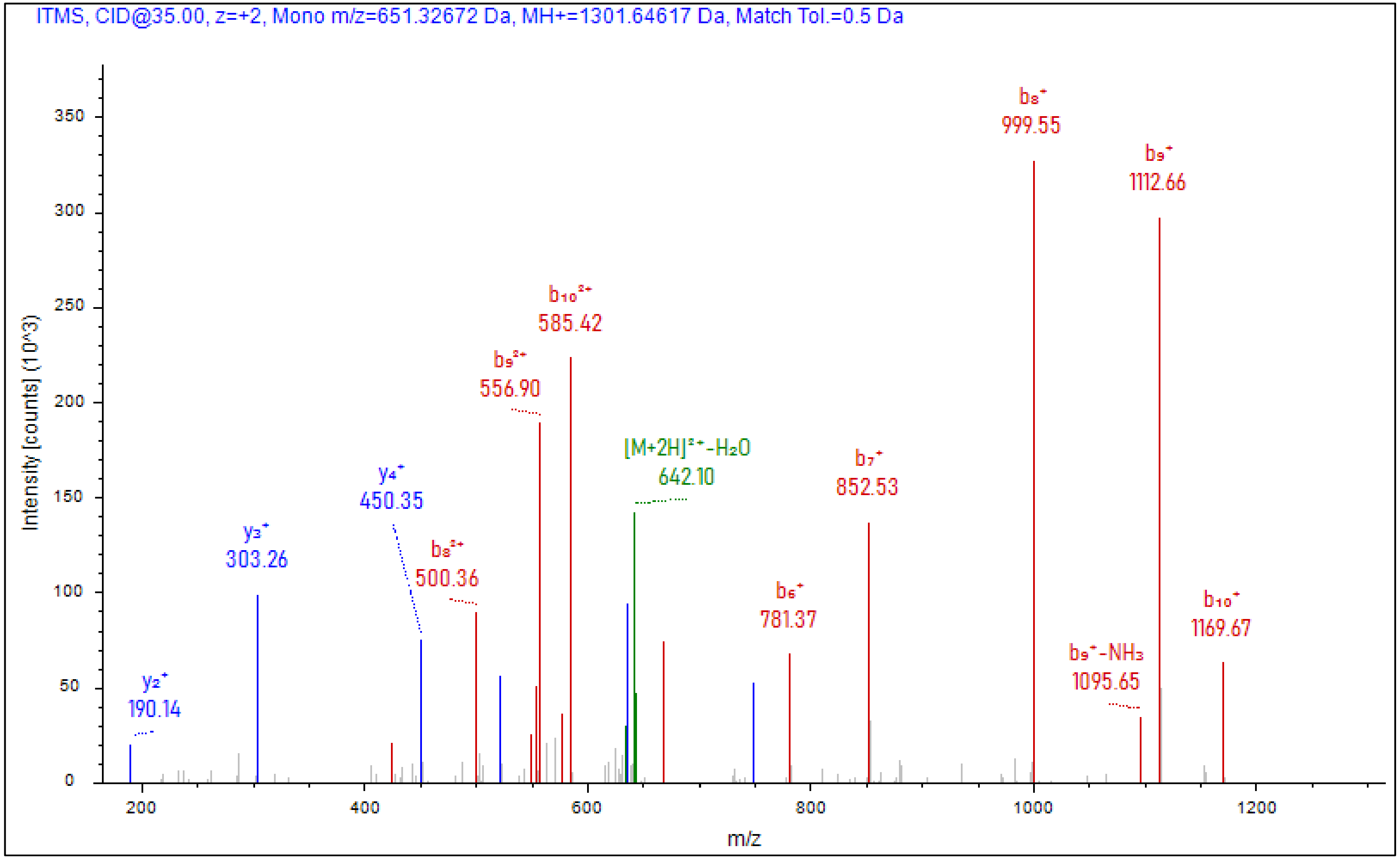

F5 Sequence: LLALGGKPVATMSGSLR, Charge: +2, Monoisotopic m/z: 835.98468 Da (-0.1 mmu/-0.12 ppm), MH+: 1670.96208 Da, RT: 39.03 min, Identified with: Mascot (v1.30); IonScore:83, Exp Value:8.4E-009, Ions matched by search engine: 21/156

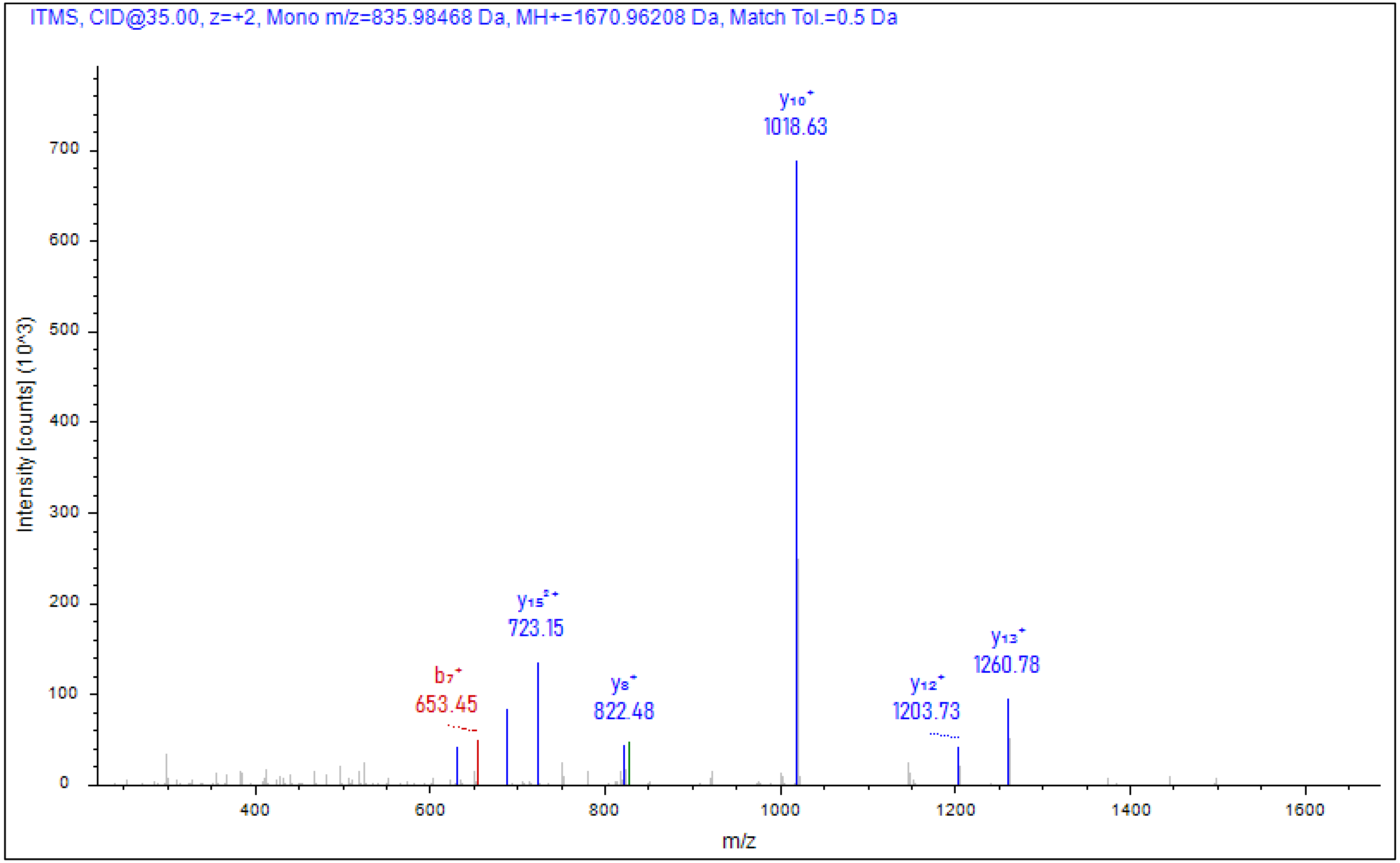

F6 Sequence: MVAALVNDFTLIIGELK, Charge: +2, Monoisotopic m/z: 924.02399 Da (+2.95 mmu/+3.2 ppm), MH+: 1847.04070 Da, RT: 68.55 min, Identified with: Mascot (v1.30); IonScore:99, Exp Value:1.9E-010, Ions matched by search engine: 14/162

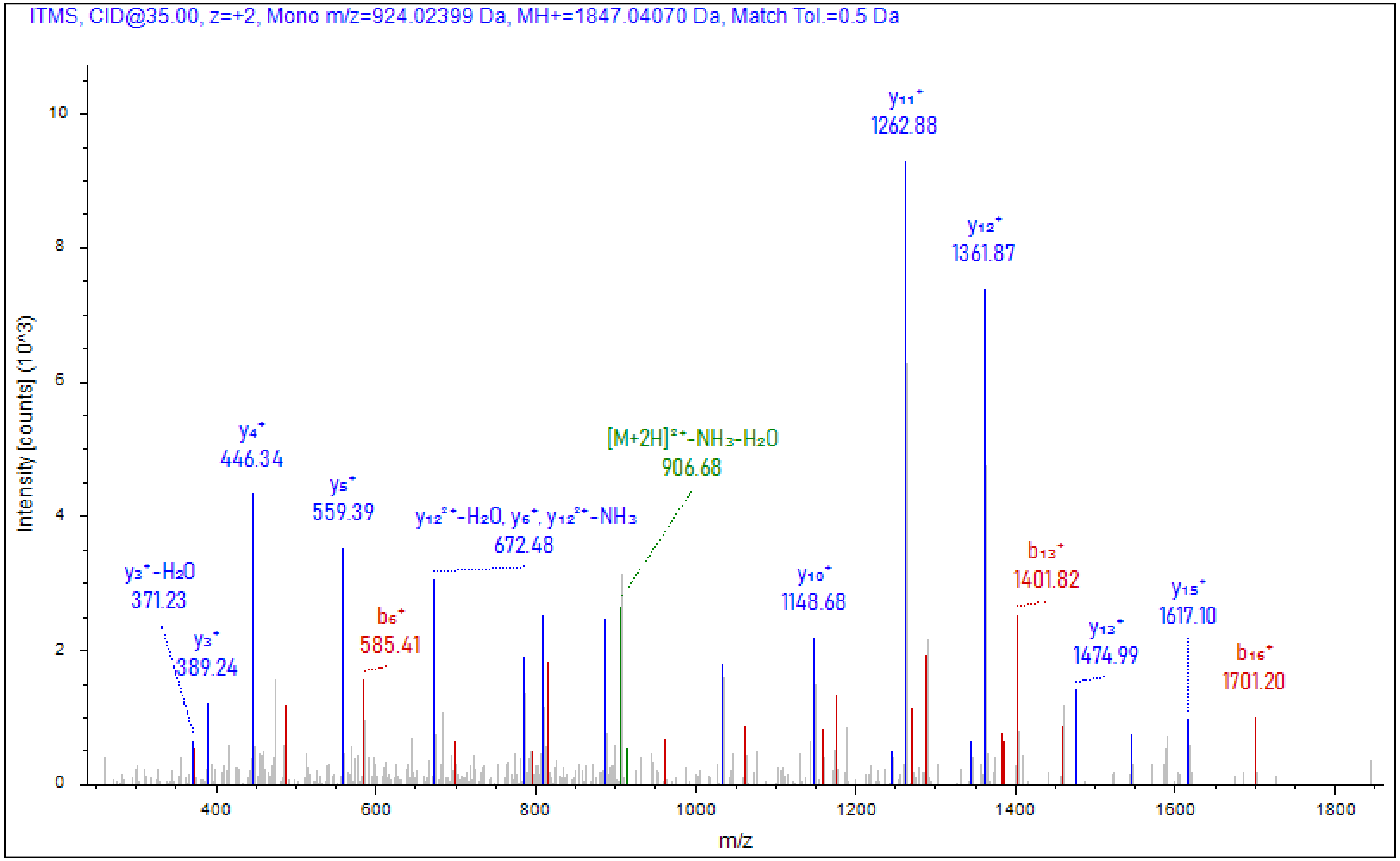

F7 Sequence: FEELYTEAALHVDALAER, Charge: +2, Monoisotopic m/z: 1039.01477 Da (-0.74 mmu/-0.72 ppm), MH+: 2077.02226 Da, RT: 63.26 min, Identified with: Mascot (v1.30); IonScore:99, Exp Value:3.0E-010, Ions matched by search engine: 13/166

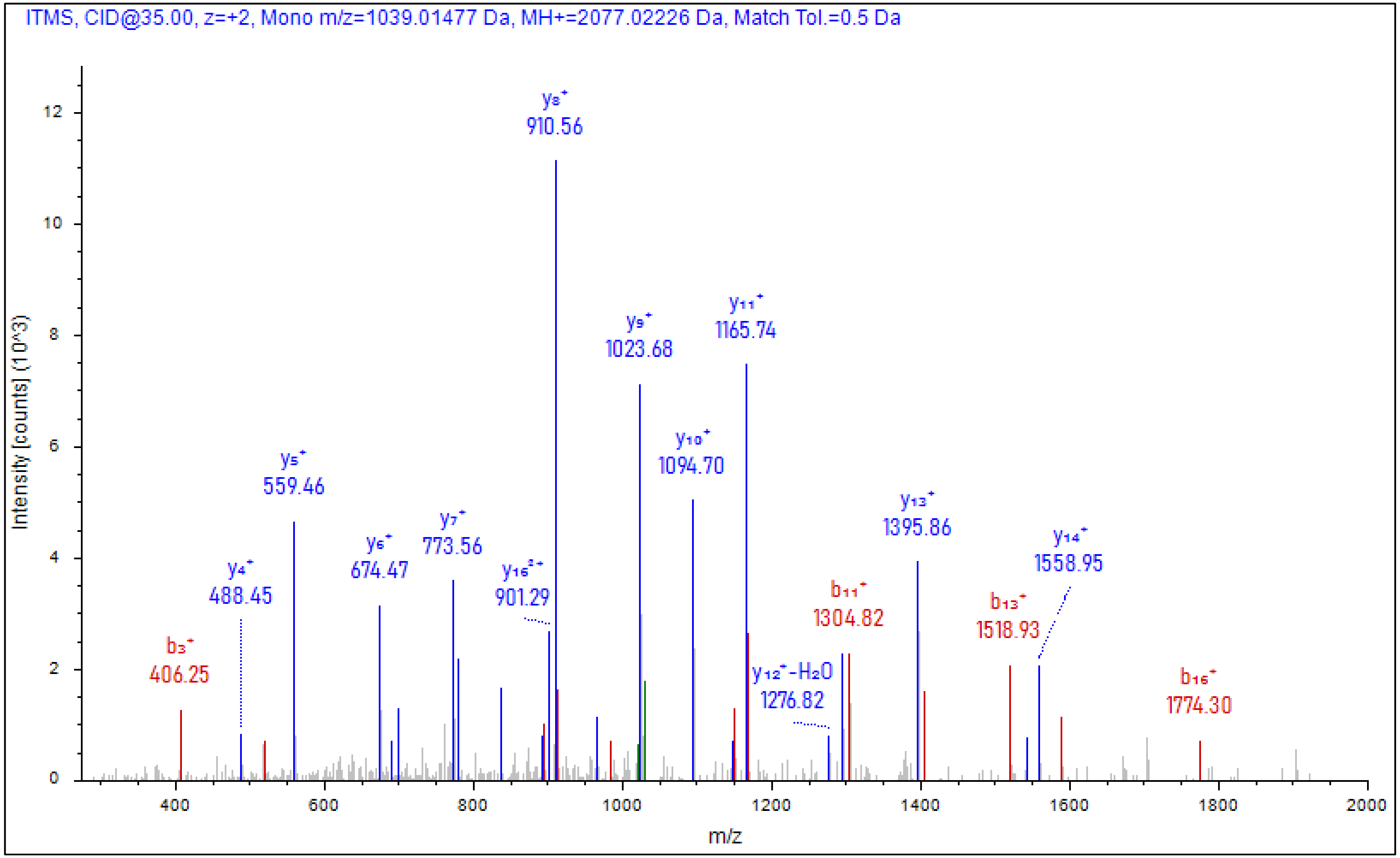

F8 Sequence: SGMKYAESVQDETTGDLLLAIHSSLEK, Charge: +2, Monoisotopic m/z: 2936.46025 Da (+0.99 mmu/+0.78 ppm), MH+: 2519.25322 Da, RT: 58.01 min, Identified with: Mascot (v1.30); IonScore:140, Exp Value:2.2E-014, Ions matched by search engine: 14/248

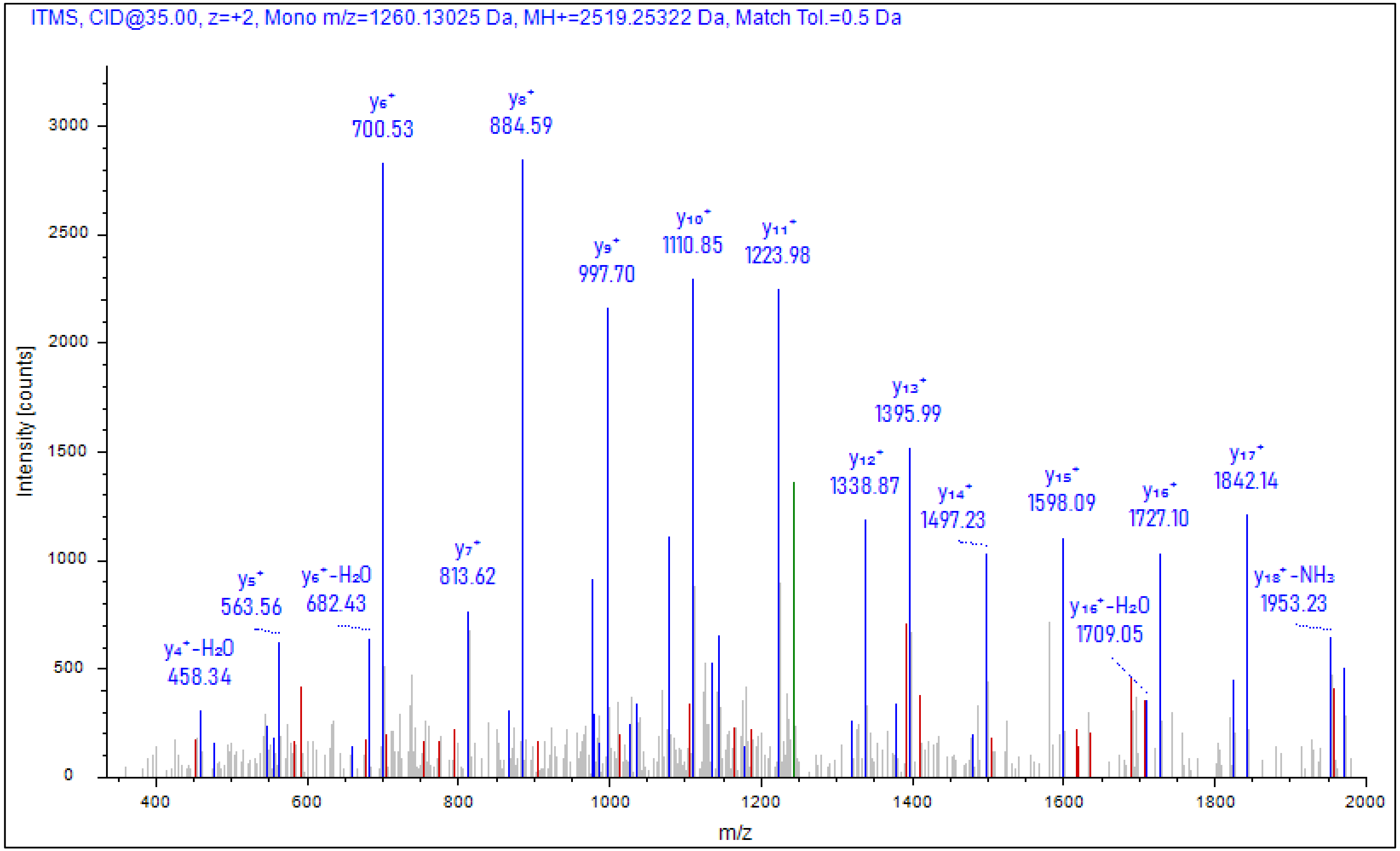

Supplement: Supplementary file 1 [file molecules-24-02516-s001.pdf]
